# The Position of the Hospital Saturday Fund

**Published:** 1920-05-15

**Authors:** Philip A. Inman

**Affiliations:** Secretary. Head Office: 54 Gray's Inn Road, London, W.C. 1.


*To the Editor of* The Hospital.

### The Position of the Hospital Saturday Fund.

*To the Editor of* The Hospital.

Sir,—My attention has been directed to a paragraph appearing in The Hospital of the 1st instant, under the heading "The Cup-Tie Programme," in which references are made to the "Hospital Saturday Fund." As certain statements made therein may lead to an erroneous impression, I venture to think that a brief record of the Fund's activities would not be out of place.

Collections are made week by week in 20,000 Metropolitan workshops, factories, and business houses, and it is estimated that 400,000 people are regular subscribers to these collections. The honorary workers are numbered by the thousand, whilst, in addition, there are thirty-three strong and active local committees working enthusiastically in and around the City of London. These figures scarcely warrant the assertion that "the Fund touches but a very small percentage of the works and workshops of London." That there are still opportunities for advancement no one will deny, and, if the progress made during recent years is studied it will be found that these opportunities are not being neglected. In the year 1914 the income was £40,676, in 1919 it reached £74,848, whilst during the first three months of the current year the amount received exceeds that of the coresponding period of last year by £3,000.

It is further stated in the paragraph under review "If the total sum collected by the Fund is compared with the number of 'workers' in London it will be seen how few contribute to it." I have already referred to the splendid support given to the Hospital Saturday Fund by the "workers," but it must also be borne in mind that the employés of a large number of firms contribute direct to their local hospital. The Fund does not interfere with any such collections, for whilst it is anxious to extend its activities, it does not desire to progress to the disadvantage of any individual hospital.

The *raison d'être* of the paragraph in question was the Cup-tie football match at Stamford Bridge. There were 50,000 spectators present on that occasion, and a large majority of them came from Birmingham, Huddersfield, and other provincial places. "Most of them," therefore, were entirely outside the scope of the Fund. It was quite possible that the London spectators are contributors towards the amount which is annually received from the supporters of the Chelsea Football Club.

I am, yours faithfully,

Philip A. Inman, Secretary.

Head Office : 54 Gray's Inn Road, London, W.C. 1.

May 4, 1920.

[We gladly publish Mr. Inman's letter. It raises more than one point of great importance at the present moment. We shall therefore return to the subject in our next issue.—Ed. The Hospital.]

